# Bullosis Diabeticorum

**DOI:** 10.5811/westjem.2016.1.29710

**Published:** 2016-03-02

**Authors:** Meina J. Michael, Jason M. Mefford, Shadi Lahham, Carrie E. Chandwani

**Affiliations:** * The George Washington University School of Medicine and Health Sciences, Washington, DC; † University of California Irvine Medical Center, Department of Emergency Medicine, Orange, California

## CASE

A 63-year-old female with insulin-dependent type II diabetes mellitus and end-stage renal disease presented to the emergency department with spontaneous blistering to the tips of her left index and middle fingers. The blisters had gradually become tense and mildly painful over the preceding 10 days. She denied burn injury, trauma, fever, or new medications. On physical exam, the patient was noted to have a tense, nontender bullae on the pad of the left middle finger, and a collapsed, hemorrhagic bullae on the left index finger. There were no signs of inflammation or infection. A radiograph of the left hand, complete blood count, and basic metabolic panel were unremarkable. The diagnosis of bullosis diabeticorum was made, and supported by a consulting endocrinologist.

## DISCUSSION

Bullosis diabeticorum is a rare cutaneous complication in those with diabetes mellitus. The condition was first recognized in 1930, and the name coined in 1967. It usually arises in those with longstanding diabetes, and affects 0.5% of the diabetic population in the U.S. in a 2:1 male-to-female ratio.^1^ It erupts spontaneously mainly on acral surfaces of the upper and lower extremities, but may also involve the trunk. The exact etiology is not fully known, but thought to involve poor vascular supply and increased venous pressure that leads to splitting of the dermal-epidermal junction at the level of the lamina lucida.^2^ It has not been shown to be related to level of glycemic control. These lesions may be confused with a burn injury; however, they only require supportive management and usually heal without intervention in 2–6 weeks, though affected areas should be monitored for secondary infection.^3^

## Figures and Tables

**Figure:**
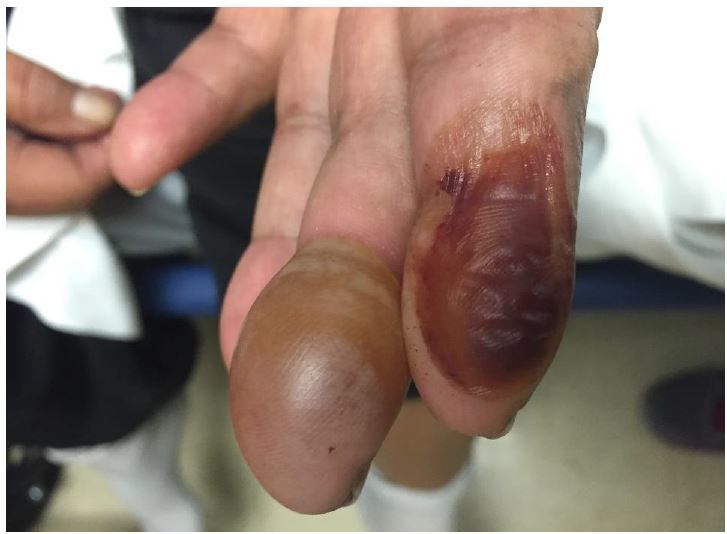
Bullosis diabeticorum of the fingertips.
